# Antitumor Mechanism of the Essential Oils from Two Succulent Plants in Multidrug Resistance Leukemia Cell

**DOI:** 10.3390/ph12030124

**Published:** 2019-08-26

**Authors:** Paola Poma, Manuela Labbozzetta, James A. McCubrey, Aro Vonjy Ramarosandratana, Maurizio Sajeva, Pietro Zito, Monica Notarbartolo

**Affiliations:** 1 Department of Biological, Chemical and Pharmaceutical Science and Technology (STEBICEF), University of Palermo, 90128 Palermo, Italy; 2 Department of Microbiology and Immunology, Brody School of Medicine at East Carolina University, Greenville, NC 27858, USA; 3 Department of Plant Biology and Ecology, University of Antananarivo, P.O. Box 906, Antananarivo 101, Madagascar

**Keywords:** acute myeloid leukemia cell, *Cyphostemma juttae*, essential oil, *Kalanchoe beharensis*, multidrug resistance, NF-κB

## Abstract

Drug resistance remains a major challenge in the treatment of cancer. The multiplicity of the drug resistance determinants raises the question about the optimal strategies to deal with them. Essential oils showed to inhibit the growth of different tumor cell types. Essential oils contain several chemical classes of compounds whose heterogeneity of active moieties can help prevent the development of drug resistance. In the present paper, we analyzed, by gas chromatography-mass spectrometry the chemical composition of the essential oil of the leaves of *Kalanchoe* *beharensis* obtained by hydrodistillation and compared the chemical composition of its essential oil with that of *Cyphostemma juttae*. Our results demonstrated the anticancer and proapoptotic activities of both species against acute myeloid leukemia on an in vitro model and its multidrug resistant variant involving NF-κB pathway. The essential oils of both species produced a significant decrease in many targets of NF-κB both at mRNA and protein levels. The results corroborate the idea that essential oils may be a good alternative to traditional drugs in the treatment of cancer, especially in drug resistant cancer.

## 1. Introduction

Clinical multidrug resistance is a multifactorial and heterogeneous process that strongly limits the efficacy of antitumor drugs. Drug resistance, especially in its multiple type (MDR), remains a major challenge in the treatment of cancer. Resistance can develop by numerous mechanisms, including decreased drug uptake, increased drug efflux, activation of detoxifying systems, activation of DNA repair mechanisms, evasion of drug-induced apoptosis, elevated autophagy, and/or altered drug metabolism [1]. One of the determining causes is the overexpression of multidrug efflux transporters, such as P-glycoprotein (P-gp), Multidrug Resistance Related Proteins (MRPs) or Breast Cancer Resistance Protein (BCRP) [2,3]. Moreover, loss of pro-apoptotic factors (e.g., p53 or Bax) or overexpression of anti-apoptotic factors, such as Bcl-2 or inhibitors of apoptosis proteins (IAPs) may interfere with the induction of tumor cell killing determining the inefficacy of anticancer drugs [4]. More recently, the constitutive expression of NF-κB factor was also related to MDR [5]. In acute myeloid leukemia (AML), constitutive NF-κB has been detected in more than 50% of cases, enabling leukemic cells to resist apoptosis and stimulate uncontrolled proliferation [6]. Therefore, NF-κB is considered as a poor prognostic factor in different types of cancer, and its inhibition can lead to pro-apoptotic signal activation and pro-survival response suppression [7,8]. All these observations demonstrate the importance of NF-κB as a therapeutic target [9]. The multiplicity of the drug resistance determinants raises the question about the optimal strategies to deal with them. There is a need for new agents that are more effective and able to bypass resistance with respect to conventional ones. In this context, essential oils (EOs) inhibit the growth of different tumor cell types. EOs contain several chemical classes of compounds whose heterogeneity of active moieties can help avoid the development of resistances. Previous works demonstrated that EOs, among others, have antitumor activities on different types of tumors, such as hepatocellular carcinoma, triple negative breast cancer [10,11] and acute myeloid leukemia (AML) [12] all characterized by scarce responsiveness to chemotherapeutic drugs.

The genus *Kalanchoe* Adanson (Crassulaceae) contains approximately 150 species distributed mainly in Madagascar, South Africa, eastern Africa, and Asia. *Kalanchoe* can have diverse forms, such as shrubs, small trees, lianas and small epiphytes both having succulent leaves [13,14]. Half of *Kalanchoe* species are native to Madagascar [15]. *Kalanchoe* plants have had a long history in folk medicine, and according to Akulova-Barlow [16], some have been called “miracle leaf” for their remarkable healing properties. Cytotoxic activity was tested in tumor cell lines by Yamagishi [17] and Shirobokov [18] and indicated that the juice of 12 species has antiviral or neutralizing activity against viruses. A review by Milad et al. [19] reported that *Kalanchoe* spp. have several pharmacological activities, for example: Antiviral, sedative, antiulcer, immunomodulatory, antileishmanial, CNS depressant, anti-inflammatory, thyroid peroxidase inhibitor, cytotoxic, hepatoprotective, antioxidant, analgesic, anticonvulsant, antimicrobial, B cell development inhibitor, cardiovascular activity, antihyperglycemic, larvicidal and insecticidal. In contrast to the extensively studied herbaceous *Kalanchoe* species, *Kalanchoe beharensis* is a large evergreen shrub up to 3 m tall of the xerophytic bush in southern Madagascar [20,21]. The leaves of the plant locally known as “mongy” are used in traditional medicine as a laxative [22,23], and occasionally consumed by the ring-tailed lemur [24,25].

**Figure 1:**
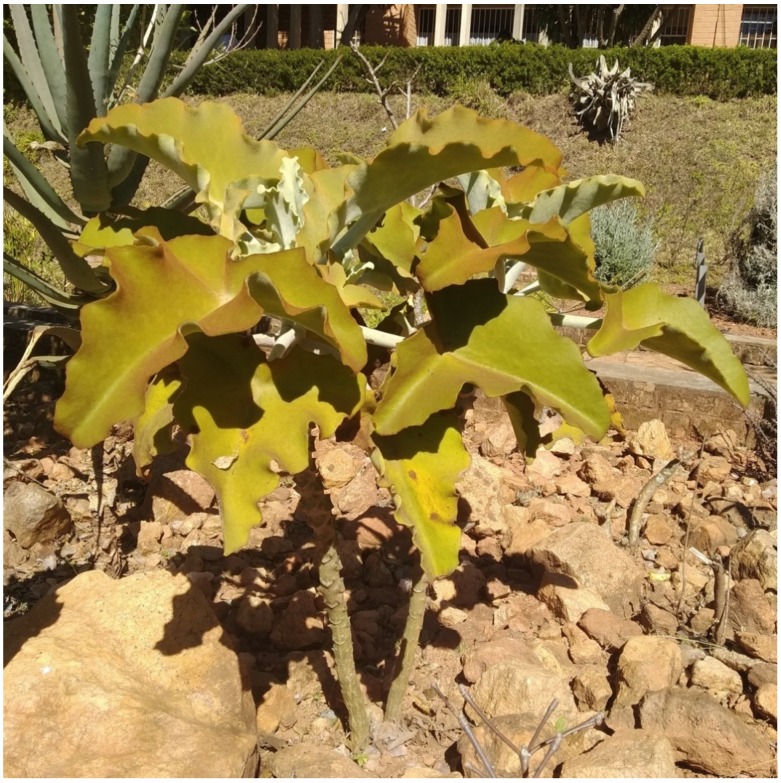
*Kalanchoe beharensis* cultivated at the Botanical Garden of the Department of Plant Biology and Ecology, University of Antananarivo (Photo V. Ramarosandratana).

From the chemical point of view, *Kalanchoe* spp. are composed of flavonoid glycosides, anthocyanins, coumarins, bufadienlolides, triterpenoids, phenanthrenes, sterols, fatty acids and kalanchosine dimalate salt [19]. However, the single chemical study was done by Ghaly et al. [26], which reported the isolation of five different flavonoids from the methanolic extracts of *K. beharensis* (Figure 1). To the best of our knowledge, there are no papers published on EO in the whole genus *Kalanchoe*.

The genus *Cyphostemma* (Planch.) Alston (Vitaceae) includes about 150 species distributed in eastern and southern Africa and Madagascar, and some species are used in traditional medicine [27]. The antiproliferative effects of solvent extracts of some *Cyphostemma* spp. on HepG2 cell line have been reported by Opoku et al. [28]. Recently, Zito et al. [11] demonstrated that the EO of *Cyphostemma juttae* has antitumor activities in triple negative breast cancer cells (MDA-MB-231, SUM 149).

In the present paper we analyzed, by gas chromatography-mass spectrometry (GC-MS), the chemical composition of the essential oil of the leaves of *Kalanchoe beharensis* obtained by hydrodistillation (HD) and compared the chemical composition of its EO with that of *Cyphostemma juttae*. We also evaluated if the cytotoxic activity of both species against AML in vitro model and its MDR variant involved NF-κB pathway. Previous papers [29,30] highlighted that HL-60R cells are characterized by an overexpression of NF-κB, P-gp and IAPs. In particular, P-gp and IAPs are targets of NF-κB and are also involved in drug and apoptosis resistance. IAPs, which are endowed with different activities, have also shown a remarkable ability to block apoptosis induced by a wide spectrum of non-related triggers, including different anti-tumor agents [30,31]. For these reasons, we selected these NF-κB target genes to investigate their expression after EOs treatment.

## 2. Results

### 2.1. Chemical Composition

The essential oil yield was 15.21 mg (0.003%). In the essential oil of *Kalanchoe beharensis*, we identified 57 compounds (87% of the whole composition) belonging to 14 different classes and functional groups of compounds (Table 1). Only compounds which had mass spectral similarity ≥80% with respect to our libraries were considered.

**Figure 2:**
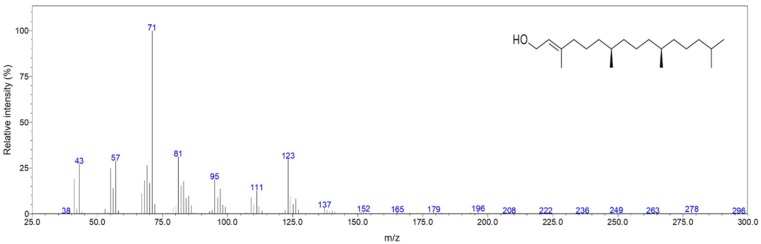
Mass spectrum and chemical structure of phytol found in the present study.

Overall, the composition was dominated by diterpene alcohols with 35.6%, followed by aliphatic alkanes (14.3%), aliphatic alcohols (11.1%) and sesquiterpene hydrocarbons (10.7%). The most abundant compounds (≥ 5%) were phytol (35%; Figure 2), tetradecanoic acid (5.1%), (*Z*,*Z*,*Z*)-9,12,15-octadecatrien-1-ol (5.1%), and heptacosane (5%) contributing together 50.2% of the total composition. Eleven compounds were detected in relative amounts between 1 and 5%, whereas 42 compounds were found in amounts < 1% (Table 1).

**Figure 3:**
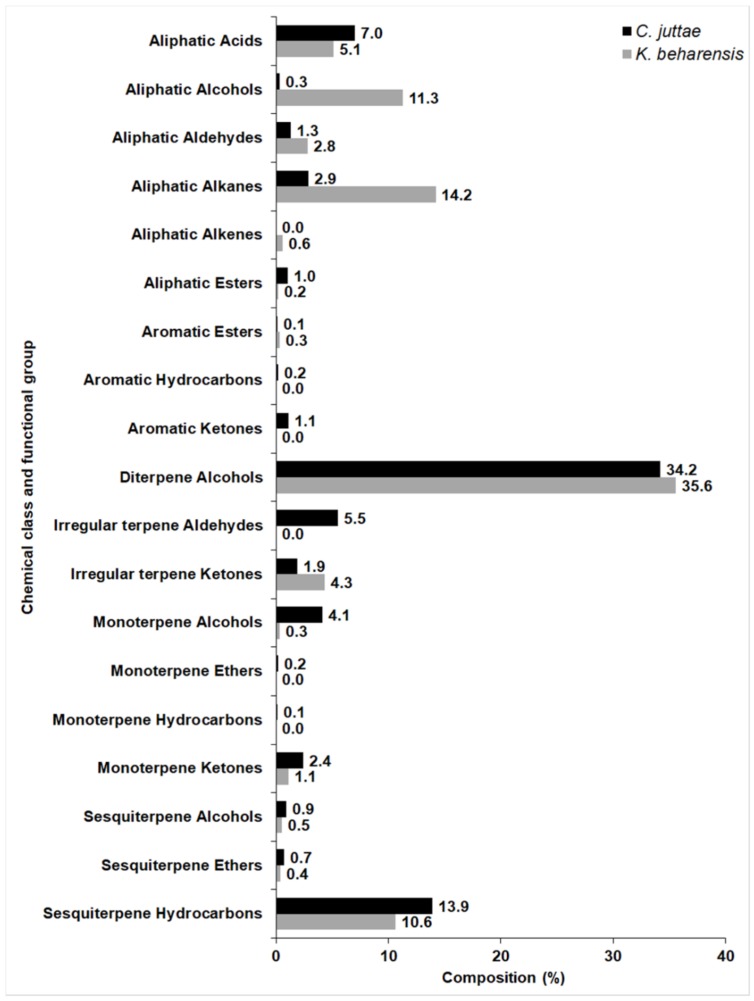
Relative (%) amounts of each chemical class and the functional group identified in *Cyphostemma juttae* and *K. beharensis.*

Zito et al. [11] found that the EO of *C. juttae* contained 34.2% of phytol and its isomer, while in the present study, *K. beharensis* contains 35% of phytol. Interestingly, also sesquiterpene hydrocarbons were present in both species with high percentages (10.6% in *K. beharensis* and 13.9% in *C. juttae*) (Figure 3).

### 2.2. Cytotoxic Effects of C. juttae and K. beharensis Essential Oils

**Figure 4:**
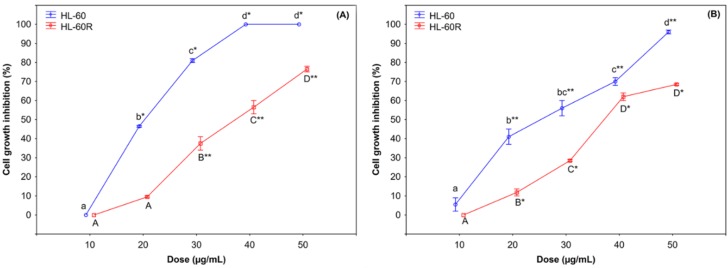
Cytotoxic activity of *C. juttae* (**A**) and *K. beharensis* (**B**) essential oils (Eos) on leukemia HL-60 and HL-60R cell lines. Cell viability was assessed by MTS. Data are expressed as mean ± standard error of at least three different experiments performed in triplicate. Different letters represent significant differences (Tukey test) in cytotoxic activity among the concentrations of each cell line (*C. juttae*: HL-60 *p* < 0.01 and HL-60R *p* < 0.05; *K. beharensis*: HL-60 *p* < 0.05 and HL-60R *p* < 0.01). Differences when treatments are compared to the controls: * *p* < 0.05; ** *p* < 0.01.

Cell growth inhibition assays revealed that the cytotoxic activity of *C. juttae and K. beharensis* essential oils on HL-60 cell line and on its MDR variant HL-60R cell line is quite equivalent. As shown in Figure 4A,B after 72 h of treatment, both essential oils induced cell growth inhibition at concentration-dependent way in the two cell lines; in Table 2 are reported the IC_50_ of *C. juttae* and *K. beharensis* essential oils. The treatment of the cell lines with doxorubicin, which is a conventional chemotherapeutic agent used as a first line treatment in AML, caused a cytotoxic effect with IC_50_ values of 8 ± 0.4 ng/mL and 1.2 ± 0.3 μg/mL for HL-60 and HL-60R, respectively. The IC_50_ values of EOs in both cell lines are very similar, while as expected doxorubicin must be present at very high concentrations to obtain IC_50_. For this reason, we suppose that EOs were not substrates of P-gp as doxorubicin is.

**Figure 5:**
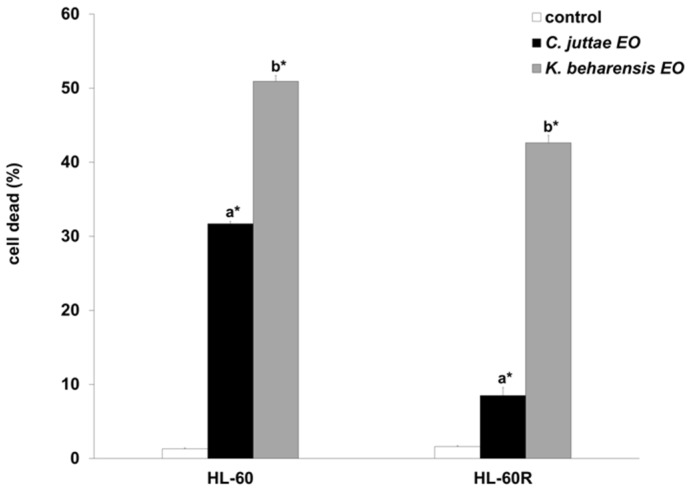
Induction of cell death by *C. juttae* or *K. beharensis* oils in HL-60 and HL-60 R cells.

**Figure 6:**
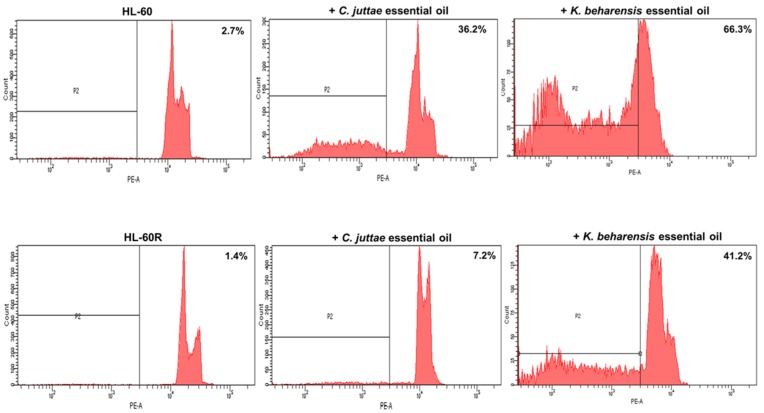
A representative example of flow cytometry analysis of cell death and cell cycle in the HL-60 and HL-60 R cells treated with 40 μg/mL concentrations of *C. juttae* or *K. beharensis* essential oils.

The results on cell death were confirmed by flow cytometry assessments (Figure 5). The HL-60 and HL-60R cell lines were incubated with the essential oils (40 μg/mL) of *C. juttae* or *K. beharensis* for 24 h, and thereafter, cell death was evaluated by flow cytometry analysis of cell DNA stained with propidium iodide. The results are in agreement with the cytotoxicity data, highlighting that both oils are capable of inducing cell death even in MDR variant cell line. In particular, the essential oil of *K. beharensis* caused a marked block in the preG_0_-G_1_ position comparable in HL-60 and HL-60R cells (Figure 6).

The cells were incubated with 40 μg/mL concentrations of *C. juttae* or *K. beharensis* essential oil for 24 h and thereafter cell death was evaluated by flow cytometry analysis of cell DNA stained with propidium iodide. Data are the mean ± standard error of three separate experiments. Different letters represent significant differences in cytotoxic activity among the two essential oils of each cell line (HL-60 *p* < 0.01; HL-60 R *p* < 0.01). *Differences when treatments are compared to the control *p* < 0.01 (Tukey test).

The figure shows the profiles of propidium iodide stained DNA. Numbers in the panels indicate the % of the events in the preG_0_-G_1_ position.

### 2.3. Effects of Essential Oils on NF-κB (p65 subunit) Pathway in HL-60/HL-60R Cells

In order to evaluate if *C*. *juttae* and *K. beharensis* essential oils could interfere on NF-κB DNA binding capacity, HL-60 and HL-60R cell lines were treated with the two essential oils at a concentration of 40 μg/mL for 24 h.

**Figure 7:**
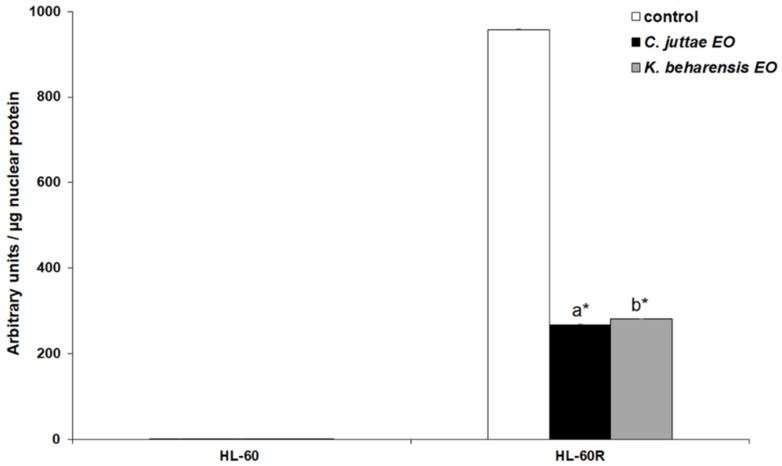
NF-κB (p65 subunit) DNA binding capacity in nuclear extracts of HL-60 and HL-60R cells.

According to the results previously published [29], the HL-60 cells showed a very slight DNA binding capacity of the p65 subunit. Otherwise, the HL-60R cells showed remarkable levels of the activated p65 subunit and both essential oils of *C*. *juttae* and *K. beharensis* determined a considerable decrease of its binding capacity to the corresponding DNA consensus sequence (Figure 7).

The cells were treated for 24 h with *C. juttae* or *K. beharensis* essential oils (40 μg/mL). Results (mean ± standard error of two experiments carried out in duplicate) are expressed as arbitrary units/μg protein of cells nuclear extracts. Different letters represent significant differences (*p* < 0.05) among the two essential oils. *Differences when treatments are compared to the control *p* < 0.01 (Tukey test).

**Figure 8:**
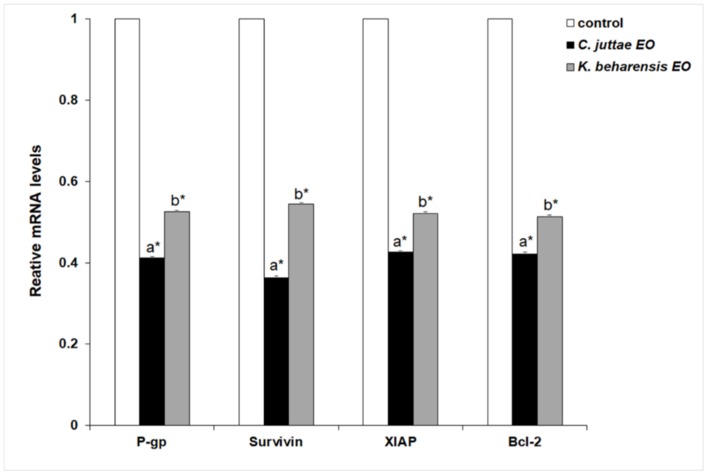
Genes mRNA expression levels by quantitative polymerase chain reaction in HL-60 R cells.

**Figure 9:**
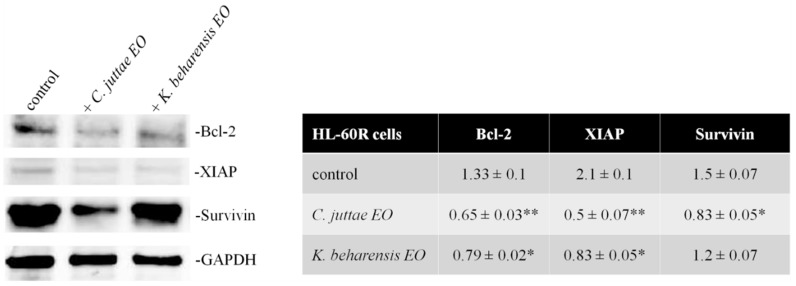
Western blotting analysis of the levels of Bcl-2, XIAP and Survivin, in HL-60 R cells treated for 24 h with *C. juttae* or *K. beharensis* essential oils (40 μg/mL). On the left the results of a representative experiment; on the right, the results expressed as mean ± standard error of two different experiments. Differences when treatments are compared to the control: * *p* < 0.05; ** *p* < 0.01.

Given these results, we investigated if the treatment with essential oils at the same conditions also modified the expression of some NF-κB targets, at mRNA and protein levels in the MDR variant cell line. The most considerable result indicated a strong reduction in some IAPs as survivin and XIAP, Bcl-2 and P-gp expression by both essential oils (Figure 8). These results are also confirmed at protein levels; essential oils of *C*. *juttae* and *K. beharensis*, in fact, produced a strong reduction of the three anti-apopototic proteins, more evident for *C*. *juttae* EO (Figure 9).

The cells were treated for 24 h with *C. juttae* or *K. beharensis* essential oil (40 μg/mL). Data are expressed as mean ± standard error of two different experiments. Different letters (a, b) represent significant differences (*p* < 0.01) among the two essential oils for each gene. *Differences when treatments are compared to the control; *p* < 0.01.

## 3. Discussion

Terpenes are known as molecules with high biological activities, and there is a wide literature as regards to phytol activities [33]. These compounds are widely present in plants and play a key role in their constitutive defenses [34]. The chemical composition of the two species prompted us to verify if their EOs have comparable activity in the human AML cell line, HL-60, and its multidrug resistant, P-gp over-expressing variant, HL-60R. The two essential oils caused cytotoxicity in HL-60 and noteworthy also on HL-60R cells, regarding cell growth inhibition and induction of cell death. The variant HL-60R was obtained treating HL-60 cells with doxorubicin (for details see Material and Methods section), and its molecular characterization was carried out previously [29,30]. This is an important model of multidrug resistant cancer because HL-60R exhibits resistance to apoptosis induced from numerous drugs, substrates of P-gp, and also from other molecules not related to the multidrug transporter. Furthermore, HL-60R overexpress many IAPs and on the contrary of their parental cell line, HL-60, contained and overexpress p65 subunit that is fundamental to form active transcriptional factor NF-kappaB. The role of this transcriptional factor is well confirmed in numerous types of cancer [35], related to all phases of cancer develop as tumorigenesis, progression, invasion and metastasis, and its overexpression can cause the aberrant expression of the protein responsible of multidrug resistance [5,36]. For these reasons, NF-κB is often studied as a potential target for the treatment of malignant tumors [37,38,39], including AML [40] for which the therapeutic choice is restricted to few anti-blastic drugs towards which cancer cells develop early resistance. Most recently, new research is being conducted towards checkpoint inhibitors and cancer immunotherapy, like CAR T-cell therapy, but the side effects are multiple and sometimes characterized by unacceptable toxicity [41]. For these reasons, the need for good alternative therapies is urgent. Furthermore, the phenomenon of acquired resistance narrows even more therapeutic possibilities. The most common mechanism for acquisition of resistance is the expression of energy-dependent transporters as ATP binding cassette (ABC) transporters, characterized by homologous ATP-binding, and large, spanning transmembrane domains, including P-gp [1]. In light of this evidence, appears even more significant our result about inhibition of NF-κB activation from essential oils in HL-60 R cells. To further confirm that the transcriptional factor can be an effective pharmacological target, we demonstrated that essential oils produced a significant decrease in many targets of NF-κB both at mRNA and protein levels. These targets are just those responsible for resistance to apoptosis induced from drugs (IAPs) and the multidrug transporter, P-gp. We have already reported that, in comparison to HL-60, HL-60R cells exhibit an increased expression of some IAP family members [29,30]. However, different factors involved in apoptosis, such as IAPs can be altered in cancer cells, thereby rendering them less prone to drug-induced cell death. Our results demonstrated that the EOs inhibited the growth and induced cell death by the suppression of signaling of the transcription factor NF-κB and by the suppression of IAP family proteins and the antiapoptotic factor Bcl-2. We propose, according to the literature [42,43] the fundamental role of NF-κB, P-gp and IAPs as possible therapeutic targets.

Defense in plants is usually constitutive, and they have mechanical or chemical defenses that can discourage and fight phytophagous insects and microorganisms attack. Drugs widely used in the treatment of several human and animal diseases are often chemicals involved in plant defense. Among plants, the chemical compounds of essential oils play a key role in constitutive defense [34,44]. It is interesting to note that the EOs of the two species investigated in the present study interfere with the NF-κB factor. NF-κB is a conservative gene evolved one billion years ago [45] which play a key role in the innate immunity of insects, among others [46]. EOs obtained from some plant species inhibit the constitutive activation of NF-κB expressed in some MDR human cell lines. This mechanism may involve NF-κB functioning in insects and may explain the selective advantage for plants to produce such compounds which may damage the innate immunity of phytophagous insects and weakening their fitness. The presence of phytol as the major compound in the EOs of *C. juttae* [11] and *K. beharensis* (present study) may indicate that this compound is responsible for the NF-κB inhibition.

## 4. Material and Methods

### 4.1. Plant Species

*Kalanchoe beharensis* Drake (Crassulaceae) is an endemic species of the xerophytic forests of southern Madagascar. This species, reaching about 3 m in height, has stems with leaves crowded at the branch tips. Since its leaves (12–35 cm long and 7–35 cm wide) are covered in a dense felt it is commonly known as Felt Plant or Elephant Ear [16].

*Cyphostemma juttae* (Dinter and Gilg) Desc. (Vitaceae) is a tree-like succulent growing in Namibia and it is adapted to dry habitat. Leaves are deciduous and are produced in Summer during the vegetative season.

### 4.2. Plant Material

Leaves of *K. beharensis* were collected in July 2018 from plants cultivated at the Botanical Garden of the University of Palermo. The plants were raised from seeds in 1984 and cultivated in the open with reference code: Crassulaceae N 39. Leaves of *C. juttae* were collected in July 2017 at the Botanical garden. The seeds of both species were obtained, and the plants raised before the Convention on Biological Diversity (CBD) entered into force on 29 December 1993, and therefore, are pre-CBD specimens. The matrices were placed in paper bags and kept at −30 °C for 24 h before hydrodistillation. No specific permits were required for the described location and for the collection of plant material because the plants are part of the living collection of the Botanical Garden of the University of Palermo and the authors have access to that.

### 4.3. Essential Oil Extraction

Leaves (507 g) were hand-cut into small pieces (~2 cm) and hydrodistilled for 3 h in a Clevenger-type apparatus, using n-pentane as collection solvent [47]. The oil was dried by anhydrous sodium sulphate and stored at −30 °C until chemical analysis and pharmacological tests. To prepare the stock solution for biological studies, 2 mg of essential oil was dissolved in 1 mL of dimethyl sulfoxide (DMSO). *C. Juttae* EO was obtained from the same batch of Zito et al. [11].

### 4.4. Gas Chromatography-Mass Spectrometry

The essential oil of *K. beharensis* was analyzed by GC-MS on a single quadrupole Shimadzu GC-MS-QP2010 Plus equipped with an AOC-20i autoinjector (Shimadzu, Kyoto, Japan) and a Supelcowax 10 capillary column (30 m long, 0.25 mm i.d., 0.25 μm film thickness) (Merck KGaA, Darmstadt, Germany). One μL of diluted samples (1/100 v/v, in n-pentane) was injected at 250 °C in a split ratio of 1:1, and the column flow (carrier gas: Helium) was set at 1 mL/min. The GC oven temperature was held for 5 min at 40 °C, then increased by 2 °C/min to 180 °C, held for 60 min and finally raised to 240 °C at 10 °C/min. The MS interface worked at 280 °C, and the ion source at 250 °C. Mass spectra was taken at 70 eV (in EI mode) from m/z 30 to 600. The GC/MS data were analyzed using the GCMSolution package, Version 4.11.

The chemical analysis of *C. juttae* was performed by GC-MS on a Shimadzu GC-MS-QP2010 Ultra equipped with an AOC-20i autoinjector (Shimadzu, Kyoto, Japan) and a ZB-5 fused silica column (30 m long, 0.32 mm i.d., 0.25 μm film thickness,) as described in detail by Zito et al. [11].

### 4.5. Identification of Compounds

Identification of compounds was carried out using the mass spectral libraries FFNSC 2, W9N11, ESSENTIAL OILS (available in MassFinder 3), and Adams [48]. We only considered compounds which were present in our digital libraries with a calculated Kovats index ± 10 compared to mass spectra and/or Kovats retention indices found in NIST11, SciFinder and Pherobase [49] database. Kovats retention indices were calculated using a series of n-alkanes (C_10_-C_30_).

### 4.6. Cell Lines and Culture Conditions

The HL-60 cells were obtained from ATCC^®^ (CCL-240, Rockville, MD, USA), while its variant HL-60R, was selected for multidrug resistance (MDR) by exposure to gradually increasing concentrations of doxorubicin. The cells were routinely maintained in Roswell Park Memorial Institute (RPMI) 1640 (HyClone Europe Ltd., Cramlington, UK) supplemented with 10% heat-inactivated fetal calf serum, 2 mM L-glutamine, 100 units/mL penicillin and 100 μg/mL streptomycin (all reagents were from HyClone Europe) in a humidified atmosphere at 37 °C in 5% CO_2_.

### 4.7. Cell Growth Inhibition Assays

The cells were seeded at 1 × 10^4^ cells/well into 96-well plates and then incubated overnight. At time 0, the medium was replaced with complete, fresh medium, and the essential oils were added in various concentrations. After 72 h, 15 μL of a commercial solution (obtained from Promega Corporation, Madison, WI, USA) containing 3-(4,5-dimethylthiazol-2-yl)-5-(3-carboxymethoxyphenyl)-2-(4-sulphophenyl)-2H-tetrazolium (MTS) and phenazine ethosulfate were added. The plates were incubated for 2 h in a humidified atmosphere at 37 °C in 5% CO_2_. The bioreduction of the MTS dye was assessed by measuring the absorbance of each well at 490 nm. Cytotoxicity was expressed as a percentage of the absorbance measured in the control cells.

### 4.8. Evaluation of Cell Death by Flow Cytometry

Cells were washed twice with ice-cold PBS and then resuspended at 1 × 10^6^/mL in a hypotonic fluorochrome solution containing propidium iodide (PI) 50 μg/mL in 0.1% sodium citrate plus 0.03% (v/v) Nonidet P-40. After 1 h of incubation in this solution, the samples were filtered through nylon cloth, 40 μm mesh, and their fluorescence was analyzed as single-parameter frequency histograms using a FACSort instrument (Becton Dickinson, Mountain View, CA, USA). The data were analyzed with CellQuest™ (Becton Dickinson, Mountain View, CA, USA). Cell death was determined by evaluating the percentage of events accumulated in the preG_0_-G_1_ position analyzed by flow cytometry.

### 4.9. NF-κB Activation

The DNA binding capacity of NF-κB (p65 subunit) was measured in the nuclear extracts of cells treated using the TransAM NF-κB and Nuclear Extract^TM^ Kits (Active Motif, Carlsbad, CA, USA) according to the manufacturer’s instructions and as previously described [11]. The results were expressed as arbitrary units: One unit is the DNA binding capacity shown by 2.5 µg of whole cell extract from Jurkat cells stimulated with 12-Otetradecanoylphorbol-13-acetate (TPA)+calcium ionophore (CI)/µg protein of HL-60/R nuclear extracts.

### 4.10. Extraction of Cellular RNA and Reverse Transcription-Quantitative PCR (RT-qPCR)

Total RNA was extracted from cell lines using TRIzol reagent (Invitrogen Life Technologies, Carlsband, CA, USA). For the evaluation of gene expression, RNA was reverse transcribed using a high capacity complementary DNA (cDNA) reverse transcription kit (Applied Biosystems Life Technologies Inc., Foster City, CA, USA). The resulting cDNAs were subjected to real-time RT-PCR using the TaqMan Gene Expression Master Mix kit (Applied Biosystems Life Technologies Inc., Foster City, CA, USA) in triplicates. The PCR cycling conditions were as follows: Denaturation at 50 °C for 2 min, annealing at 95 °C for 10 min, followed by 40 cycles of 95 °C for 15 sec and extension at 60 °C for 60 min. The running of the samples and data collection were performed on a StepOne AB Real Time PCR system (Applied Biosystems Life Technologies Inc., Foster City, CA, USA). β-actin was used as an internal standard. The specific primers used were: Survivin Hs00153353, XIAP Hs00236913, Bcl-2 Hs00236329, ABCB1 Hs00184005 (Applied Biosystems Life Technologies Inc., Foster City, CA, USA). Relative expression was calculated using the comparative Ct method [ΔCt = Ct(target gene) − Ct(housekeeping gene)]. Where Ct was the fractional cycle number at which the fluorescence of each sample passed the fixed threshold. Fluorescence was measured at 515–518 nm using StepOne AB Real Time PCR System software (Applied Biosystems Life Technologies Inc., Foster City, CA, USA). The ΔΔCt method was used to determine gene expression levels. ΔΔCt was calculated using the formula: ΔΔCt = ΔCt_(each sample)_ − ΔCt_(reference sample)_. Fold change was calculated using the 2^-ΔΔCt^ equation.

### 4.11. Western Blot Analysis

For Western blot analyses, polyclonal rabbit antibody against XIAP was obtained from Cell Signaling Technology Inc., Danvers, MA, polyclonal rabbit antibody against survivin from Abcam Limited, Cambridge, UK; mouse monoclonal antibodies against Bcl-2 were obtained from Santa Cruz Biotechnology Inc., Santa Cruz, CA, USA; monoclonal mouse antibody against GAPDH was obtained from Sigma-Aldrich Srl, Milan, Italy. Whole cellular lysates from HL-60 and HL-60 R cells were obtained using RIPA buffer (Santa Cruz Biotechnology Inc., Santa Cruz, CA, USA) and 15 µg of protein were subjected to 10% SDS-polyacrylamide gel electrophoresis. Proteins were electrophoresed onto nitrocellulose membrane (Amersham, Pharmacia Biotech, Milan, Italy) using a semi-dry fast blot apparatus (Bio-Rad, Milan, Italy). Membranes were blocked with 5% (w/v) BSA in PBS-0.1% (v/v) Tween 20 for 1 h and then probed with the anti-XIAP (1:500), anti-survivin (1:2000), anti-Bcl-2 (1:1000), and anti-GAPDH (1:25000) antibodies. Hybridization was visualized using an enhanced chemiluminescence detection kit (SuperSignal West Femto Maximum Sensitivity Substrate, Thermo Scientific Life Technologies Italia, Monza, Italy) and the Versa DOC imaging system (BioRad Laboratories, Milan, Italy). Immunoblots were quantified by densitometry and results were expressed as arbitrary units (protein/GADPH).

## Figures and Tables

**Table 1:** Essential oil composition of *Kalanchoe beharensis*. Compounds belonging to the same chemical class and functional group [32] are arranged according to Kovats Retention Indices (KRI) of the Supelcowax 10 column.

RI ^a^	Ident ^b^	Compound	Relative Amount (%)	MSS ^c^ (%)
		*Aliphatic Acids*		
2720	RI, MS	Tetradecanoic Acid	5.1	94
		*Aliphatic Alcohols*		
1354	RI, MS	Hexanol	0.2	95
1407	RI, MS	(*E*)-2-Hexen-1-ol	0.1	88
1452	RI, MS	1-Octen-3-ol	1.3	95
1558	RI, MS	Octanol	1.5	97
1616	RI, MS	(*E*)-2-Octen-1-ol	1.0	91
1661	RI, MS	Nonanol	0.1	88
1761	MS	(*Z*)-9-Tetradecen-1-ol	0.2	87
1763	RI, MS	Decanol	0.1	87
1968	RI, MS	Dodecanol	0.2	88
2174	RI, MS	Tetradecanol	0.3	83
2585	RI, MS	Octadecanol	0.4	90
2793	MS	Eicosanol	0.8	91
2816	MS	(*Z*,*Z*,*Z*)-9,12,15-Octadecatrien-1-ol	5.1	85
		*Aliphatic Aldehydes*		
1050	RI, MS	Hexanal	0.5	92
1218	RI, MS	(*E*)-2-Hexenal	0.7	95
1389	RI, MS	Nonanal	0.4	96
1494	RI, MS	Decanal	0.6	95
2023	RI, MS	Pentadecanal	0.5	90
2129	RI, MS	Hexadecanal	0.1	89
		Aliphatic Alkanes*		
1400	RI, MS, Co-GC	Tetradecane	0.1	standard
1700	RI, MS, Co-GC	Heptadecane	0.04	standard
1900	RI, MS, Co-GC	Nonadecane	0.2	standard
2200	RI, MS, Co-GC	Docosane	0.4	standard
2300	RI, MS, Co-GC	Tricosane	2.4	standard
2500	RI, MS, Co-GC	Pentacosane	3.6	standard
2600	RI, MS, Co-GC	Hexacosane	0.7	standard
2700	RI, MS, Co-GC	Heptacosane	5.0	standard
2900	RI, MS, Co-GC	Nonacosane	1.8	standard
		*Aliphatic Alkenes*		
1862	MS	(*Z*,*Z*,*Z*)-3,6,9-Tetradecatriene	0.6	87
		*Aliphatic Esters*		
1607	RI, MS	Hexyl hexanoate	0.1	86
2213	RI, MS	Methyl palmitate	0.1	88
		*Aromatic Esters*		
2164	MS	2-Ethylhexyl benzoate	0.1	93
2286	MS	2-Ethylhexyl salicylate	0.2	80
		*Diterpene Alcohols*		
2296	RI, MS	Isophytol	0.6	88
2615	RI, MS	Phytol	35.0	96
		*Irregular terpene Ketones*		
1807	RI, MS	(*E*)-*β*-Damascenone	0.2	83
2123	RI, MS	Hexahydrofarnesyl acetone	4.1	94
		*Monoterpene Alcohols*		
1789	RI, MS	*α*-Campholenol	0.1	84
2216	RI, MS	Carvacrol	0.2	91
		*Monoterpene Ketones*		
1630	RI, MS	Pulegone	0.5	95
1908	RI, MS	Piperitenone	0.6	86
		*Sesquiterpene Alcohols*		
1552	RI, MS	(*Z*)-Sesquisabinene hydrate	0.2	86
2042	RI, MS	(*E*)-Nerolidol	0.1	83
2308	MS	6-epi-Shyobunol	0.2	83
		*Sesquiterpene Ethers*		
1956	RI, MS	Caryophyllene oxyde	0.4	92
		*Sesquiterpene Hydrocarbons*		
1470	RI, MS	*α*-Copaene	0.2	94
1572	RI, MS	*β*-Caryophyllene	4.5	97
1644	RI, MS	*α*-Humulene	0.1	85
1665	MS	(*E*)-*β*-Bergamotene	0.04	83
1696	RI, MS	*γ*-Humulene	0.1	85
1736	RI, MS	*δ*-Cadinene	0.1	85
1926	RI, MS	Neophytadiene	2.8	94
1955	MS	Neophytadiene isomer ^#^	1.1	95
1982	MS	Neophytadiene isomer ^#^	1.4	95
2236	MS	Neophytadiene isomer ^#^	0.3	86

^a^ RI = Experimental for Supelcowax 10 capillary column (30 m long, 0.25 mm i.d., 0.25 μm film thickness). ^b^ Ident.: RI = NIST Standard Reference Database for polar capillary columns; MS = identification based on comparison of Mass Spectra; Co-GC = Co-injection of authentic compounds. ^c^ MSS: Mass Spectrum Similarity. ^#^ isomer not identified.

**Table 2:** IC_50_ values of the two cell lines treated with the essential oils of *C. juttae* and *K. beharensis.*

	HL-60	HL-60R
	IC_50_	IC_50_
*C. juttae* EO	22.0 ± 0.3 μg/mL	36 ± 1.2 μg/mL
*K. beharensis* EO	25.0 ± 0.6 μg/mL	36.5 ± 0.3 μg/mL
